# 

**Published:** 1853-10

**Authors:** 


### CONTENTS

OF NO IV.

### ORIGINAL COMMUNICATIONS.

### ESSAYS AND CASES.

ARTICLE I.—A Complicated Case of Dyspepsia with Hysterical Symptoms, dependent upon Displacement of the Uterus, etc. By JOHN C. DARBY, M.D., Lexington, Ky. - 277
ART. II.—French Surgery, in a letter to Professor Gross. By ELKANAH WILLIAMS, M.D., of Cincinnati. - - 283
ART. III.—A Case in which Labor was Retarded by a Large Tumor on the Nates of the Fetus. By JNO. BARTLETT, M.D., one of the Physicians to the Louisville Marine Hospital. - - - - - - 300
ART. IV.—Dysentery, and Retroversion of the Uterus considered as an effect of the same cause. By R. R. GRESHAM, M.D., Ebenezer, Miss. - - - 302
ART. V.—Red Sulphur Spring of Macon County, Tennessee—Its Medical Virtues. Communicated in a letter to Dr. YANDELL. By ARCHIBALD M. DEBOW, of Hartsville, Tennessee. - - - - - - 304

### REVIEWS.

ART. VI.—Fracture Tables. By FRANK R. HAMILTON, A.M., M.D., Professor of Surgery in the University of Buffalo and Surgeon to the Buffalo Hospital of the Sisters of Charity; with a Supplement, compiled from Dr. Hamilton's Notes, by JOHN BOARDMAN, A.B.; comprising in all an analysis of four hundred and sixty-one cases of fractures. - - - - - - 309

SELECTIONS FROM FOREIGN AND HOME JOURNALS.

On the Theories of Animal Heat. - - - - 313
On the Influence of Parasites in the production of Disease. - 320
Dysmenorrhea. - - - - - - 322
A Medical Poem. - - - - - - 323
On the Treatment of Fever by large doses of Sulphate of Quinia at St. George's Hospital. - - - - 326
Removal of a Ring from a Young Lady's Finger. - - 331
Uva Ursi a substitute for Ergot. - - - - 332
A Case of Hepatic Abscess. - - - - - 333
Spermatorrhea. - - - - - - 335
Observations on the Non-occurrence of Secondary Attacks of Dysentery. - - - - - - 337
On the Employment of Iodine Injections in Chronic Dysentery 338
Opium in Irritable and Anemic states of the Brain in Fever - 340
Prosecutions of Medical Men. - - - - 346
Iodide of Potassium as an Antidote to Mercury. - - 347
Chloroform in Rheumatism. - - - - 351
Inhalations of Chloroform in Pneumonia. - - - 353
The Microscope. - - - - - - 354
Yellow Fever in New Orleans. - - - - 355
Anesthetic Properties of Lycoperdon Proteus. - - 358

ORIGINAL INTELLIGENCE.

Health of Louisville. - - - - - 361
Death of Dr. Cochran. - - - - - 362
A New Medical Journal. - - - - - 363
Yellow Fever in the South. - - - - - 364
Yellow Fever in Philadelphia. - - - - 365
Medical Society of Kentucky. - - - - 366
Geological Survey of Kentucky. - - - - 366
Appointments in Medical Colleges. - - - - 367
Transactions of the Medical Association, New York. - - 367
Condie on Children. - - - - - 368
University of Louisville—Preliminary Course. - - 368

